# Substrate Temperature Dependent Properties of Sputtered AlN:Er Thin Film for In-Situ Luminescence Sensing of Al/AlN Multilayer Coating Health

**DOI:** 10.3390/ma11112196

**Published:** 2018-11-06

**Authors:** Liping Fang, Yidong Jiang, Shengfa Zhu, Jingjing Ding, Dongxu Zhang, Anyi Yin, Piheng Chen

**Affiliations:** Institute of Materials, China Academy of Engineering Physics, Mianyang 621700, China; fanglp26@163.com (L.F.); jiangyidong@caep.cn (Y.J.); zhushf-306@163.com (S.Z.); dingjingjing21@126.com (J.D.); zhangdongxu@caep.cn (D.Z.)

**Keywords:** luminescence sensing, aluminum nitride, erbium doping, magnetron sputtering

## Abstract

The integrity and reliability of surface protective coatings deposited on metal surface could be in-situ monitored via the attractive luminescence sensing technique. In this paper, we report the influence of substrate temperature on the properties of erbium (Er) doped aluminum nitride (AlN) film, which could be applied as a luminescent layer for monitoring the health of multilayered Al/AlN coating. The AlN:Er films were deposited via reactive radio-frequency magnetron sputtering, and the silicon substrate temperature was varied from non-intentional heating up to 400 °C. The composition, morphology, crystalline structure, and dielectric function of the AlN:Er films deposited under these different substrate temperature conditions were studied. These properties of the AlN:Er films show strong correlation with the substrate temperature maintained during film fabrication. The obtained AlN:Er films, without further annealing, exhibited photoluminescence peaks of the Er^3+^ ions in the visible wavelength range and the strongest photoluminescence intensity was observed for the AlN:Er film deposited with the temperature of substrate kept at 300 °C. The results demonstrated in this work offer guidance to optimize the substrate temperature for the deposition of AlN:Er film for future application of this sensing technique to thin metal components.

## 1. Introduction

Initially, the concept of luminescence sensing was proposed for in-situ monitoring of the health of thermal barrier coating deposited on hot components, such as metal blades, working in aggressive environments of gas turbines. An optical thin film doped with luminescent ions could be prepared under the thermal barrier coating, and luminescence could be detected when the thermal barrier coating was deteriorated by corrosion or wear [1,2,3,4,5,6].

**Figure 1:**
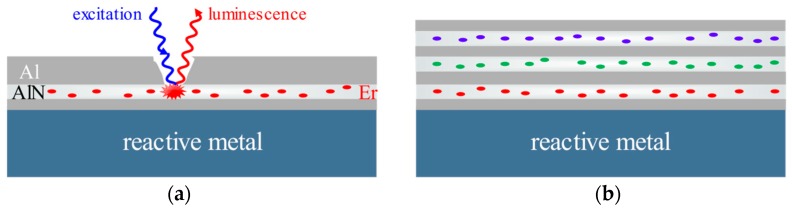
(**a**) Concept of luminescence sensing via erbium doped aluminum nitride (AlN:Er) film; (**b**) multiple luminescent layers, doped with rare-earth ions emitting at different wavelengths, could be inserted into the surface protective coating to evaluate the extent of failure.

The aim of our work is to adapt such luminescence sensing technique to monitor the health of surface protective coatings deposited on reactive metals, and the concept of this technique is shown in Figure 1a. The local failures of the surface protective coating, such as cracks and delamination sites, could be detected in-situ and non-destructively and repairing treatment could be applied to the local failures to prolong the service lifetime of the surface protective coating. This sensing technique is a promising approach for evaluating the integrity and reliability of surface protective coatings prepared on reactive metals. For such a case, multiple luminescent layers, doped with rare-earth ions emitting at different wavelengths, as depicted in Figure 1b, could be embedded in the surface protective coating to evaluate the extent of failure.

Aluminum nitride (AlN) is an attractive material, which has found wide applications in microelectronics, optoelectronics and acoustic devices, due to its wide bandgap, relatively high thermal conductivity, high electrical resistivity and high ultrasound velocity [7,8,9,10,11,12,13,14,15]. AlN has also been applied as surface protective coatings for active metals, due to its high hardness [16] and good corrosion resistance [17,18]. Al/AlN multilayered coating exhibits superior anti-wear and anti-corrosion properties, such as improved density, enhanced adhesion strength, and reduced residual stress, in comparison with the single layered Al or AlN coatings [19,20,21].

The intention of our work is to apply the luminescence sensing technique, via the deposition of a thin layer of AlN:Er film under the Al layer, to evaluate the coating health of the Al/AlN multilayer. Additionally, our previous study has demonstrated the viability of such an approach [6]. However, to apply such luminescent AlN:Er layer on thin metal components, which easily suffers from heat deformation, the substrate temperature is a key parameter that needs to be considered in the fabrication process. Nevertheless, little work has indicated the relationship between the substrate temperature and the performance of the luminescent AlN:Er films [22,23].

In this work, we investigated the effect of substrate temperature on the chemical composition, morphology, crystalline structure and the optical properties of the AlN:Er films prepared by reactive radio-frequency magnetron sputtering. Our results demonstrate that the deposited AlN:Er films without further annealing showed distinct emission peaks of the trivalent Er (Er^3+^) ions in the visible wavelength range, for all the samples prepared with substrate temperature from non-intentional heating up to 400 °C. In addition, the strongest photoluminescence intensity has been observed for the AlN:Er film prepared with substrate temperature maintained at 300 °C.

## 2. Materials and Methods

### 2.1. Film Deposition

The AlN:Er films were deposited by a customized radio-frequency sputtering system with unbalanced magnetron configuration. The diameter of the Al target was 2 inches and it was doped with 2.0 at.% of Er via arc-melting. The reason of choosing this doping level is that it provides the best photoluminescence efficiency [6,24,25]. The substrate was polished Si (100) and the working gas was a mixture of argon (70%) and nitrogen (30%). The sputtering pressure was kept at 1.0 Pa for all the samples. The sputtering pressure was kept at 1.0 Pa for all the samples. The sputtering system is in the off-axis configuration, where the sputtering target surface is not parallel to the substrate surface. The angle between the normal of the target surface and that of the substrate surface is 35°. The sputtering targets were pre-sputtered for 15 min before the shutter covering the substrate opened for deposition. The substrate-target distance was maintained at 60 mm. The power applied to the magnetron was 300 W and sputtering time was 60 min for all the samples. More experimental thin film fabrication details could be found in our previous work [6].

Five sets of AlN:Er film samples were fabricated with substrate temperature varied from non-intentional heating up to 400 °C. The substrate temperature was measured via a thermocouple attached to the back of the stainless steel substrate holder, and the deposition process of the AlN:Er films started at least 45 min after the thermal couple reads the desired temperature. For the non-heated samples, the substrates were not deliberately heated, and after 1 h deposition the temperature read below 50 °C. After deposition, no thermal annealing treatment was performed for all the samples.

### 2.2. Film Characterization

X-ray photoelectron spectroscopy technique (XPS, Physical Electronics PHI-650, Chanhassen, MN, USA) was used to characterize the chemical state and estimate the atomic ratio of the elements in the fabricated AlN:Er film. A field-emission scanning electron microscope (FESEM, FEI Helios Nanofab 600i, Lausanne, Switzerland) was used to observe the surface morphology. The root-mean-square surface roughness of the AlN:Er film was calculated based on atomic force microscopy (AFM, Bruker InSight-450 3DAFM, Billerica, MA, USA) measurements. The crystalline structure of the AlN films was characterized by grazing incidence X-ray diffraction (GIXRD, Philips X’Pert PRO, PANalytical B.V., Almelo, The Netherlands). Variable-angle spectroscopic ellipsometry (VASE, J. A. Woollam M2000, Lincoln, NE, USA) was applied to obtain the thicknesses and optical refractive indices of the thin films. The measured thickness of the AlN:Er films were in the range of 1015~1300 nm. Room temperature photoluminescence (PL) of the fabricated AlN:Er film without further annealing was recorded by a fluorescence spectrometer (Thermo Scientific DXR, Waltham, MA, USA). The film characterization details were similar to our previous work [6].

## 3. Results and Discussion

### 3.1. Chemical Composition

**Figure 2:**
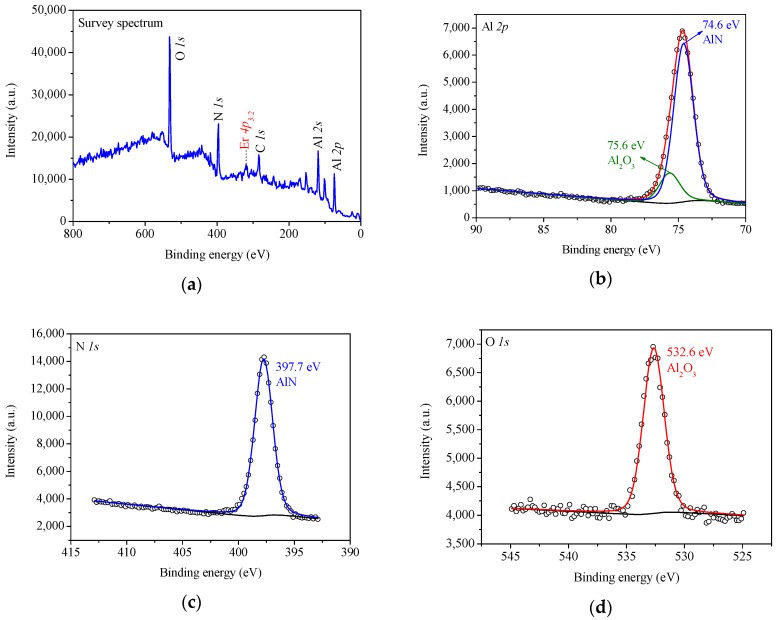
Photoelectron spectra (**a**) survey, (**b**) Al *2p*, (**c**) N *1s* and (**d**) O *1s* for a typical AlN:Er sample deposited at 400 °C after 10 min of Ar^+^ milling.

The chemical composition and the chemical state of the elements of the deposited AlN:Er films were characterized by XPS measurements. The measured spectra were calibrated by fixing the binding energy of the C *1s* photoelectron of adventitious hydrocarbon at 284.8 eV [26]. The XPS survey spectrum for a typical AlN:Er sample deposited at 400 °C is shown in Figure 2a. The photoelectron peaks corresponding to the O, N, C and Al elements were detected, and a minor peak around 320 eV corresponding to the Er *4p_3/2_* core level was also observed. The area under this peak was used to estimate the atomic concentration of Er in the AlN:Er film [27]. The high resolution Al *2p*, N *1s* and O *1s* photoelectron spectra were decomposed into the constituent components and shown in Figure 2b–d, respectively. Shirley backgrounds and Gaussian-Lorentzian spectrum types were used for the deconvolution of these elemental spectra.

As shown in Figure 2b, the curve fitting Al *2p* spectrum is decomposed into two peaks, which correspond to AlN (area ratio of 83%) and Al_2_O_3_ (area ratio of 17%), respectively. The existence of Al_2_O_3_ near the surface is possibly due to AlN hydrolysis in atmosphere [28] or oxygen leakage to the vacuum chamber during deposition. The obtained Al *2p* binding energy of Al_2_O_3_ and AlN are 75.6 ± 0.2 eV and 74.6 ± 0.2 eV, respectively. The curve fitting N *1s* spectrum illustrates that the N *1s* signal only comes from AlN, with binding energy of 397.7 ± 0.2 eV. Additionally, the curve fitting O *1s* spectrum shows that the O *1s* signal only comes from Al_2_O_3_ with binding energy of 532.6 ± 0.2 eV. These obtained binding energies of these elements are similar to literature results (see, for example, References [29,30]).

**Figure 3:**
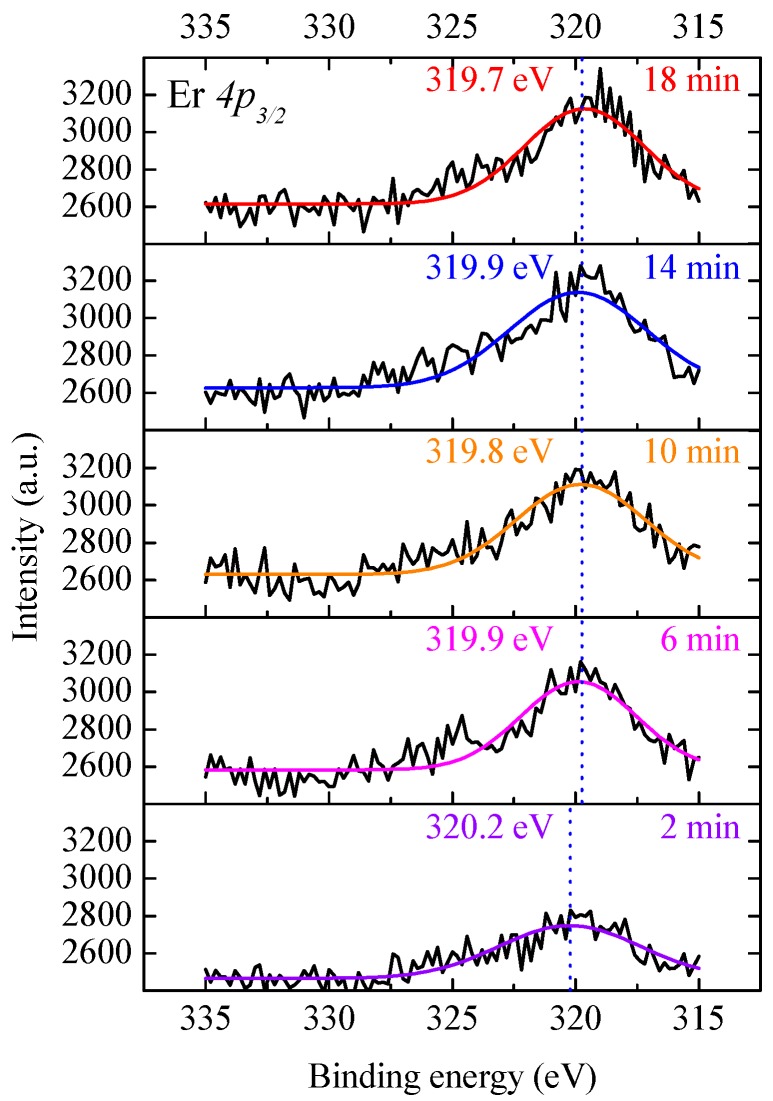
Photoelectron spectra of Er *4p_3/2_* core level for a typical AlN:Er sample deposited at 400 °C after various length of time Ar^+^ milling.

The high resolution photoelectron spectrum of the Er *4p_3/2_* core level was also recorded, and the results for a typical AlN:Er sample deposited at 400 °C after different lengths of time milling are shown in Figure 3. The spectra were fitted by Gaussian-Lorentzian functions, and the revealed peaks are shown in Figure 3. It is shown that after 2 min milling, the binding energy of the Er *4p_3/2_* core level is 320.2 ± 0.2 eV, which is almost identical to that of the Er–O bond (320.1 eV) given by Paladia et al. [31]. Additionally, after milling for more than 6 min, the spectra of the Er *4p_3/2_* core level are similar and the peaks are located at 319.8 ± 0.1 eV. These results indicate that Er–O bonds only exist near the surface of the AlN:Er film, and the chemical environment for Er is homogeneous in the bulk.

**Figure 4:**
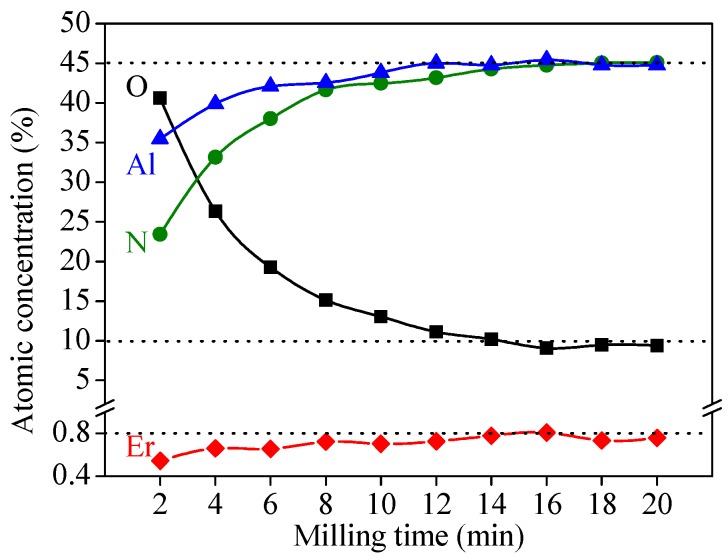
The variation of atomic concentrations with milling time.

To reveal the atomic concentration of the constituent elements of the AlN:Er films, XPS depth profiling measurements were conducted for a typical AlN:Er film deposited at 400 °C. In addition, the dependence of the atomic concentration of the constituent elements on the milling time is shown in Figure 4. To obtain the etching rate of the AlN:Er film by the Ar ions, similar depth profiling has been performed for a thin AlN:Er film of ~200 nm thick and the revealed etching rate was around 5~6 nm/min.

It is seen from Figure 4 that near the surface, the O atom has a high atomic ratio of more than 40%, this is due to the fact that the depth profiling measurement is done ex-situ and the AlN hydrolysis reaction in moist atmosphere increases the atomic ratio of O near the surface. After 12 min of milling, all the elements reach their plateaus, i.e., ~9.2% for O, ~45.0% both for Al and N, and ~0.8% for Er. Based on the results shown in Figure 4, the Er/(Er+Al) atomic ratio of the deposited AlN:Er film is calculated to be around 1.62%, which is slightly lower than that of the sputtering target (2.0%). This is due to the fact that the sputtering yield of Al is larger than Er (1.05 compared to 0.77 [32]). In addition, the Er/(Er+Al) atomic ratio of the deposited AlN:Er is found to show no dependence on the milling time, this signifies that the sputtering Al target is homogeneously doped with Er.

### 3.2. Surface Morphology

**Figure 5:**
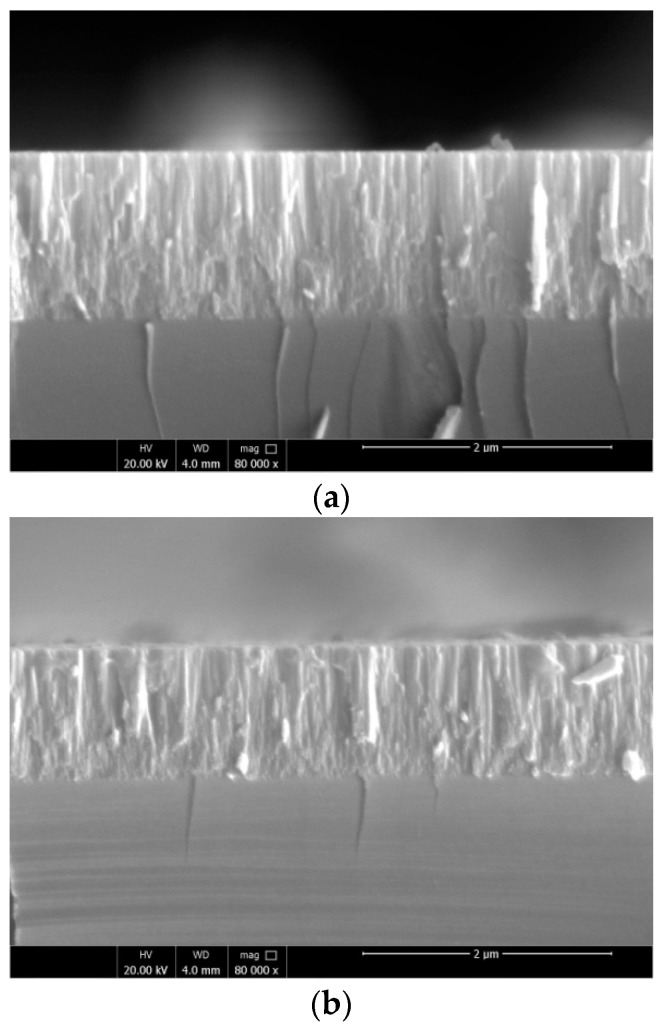
Cross-sectional morphology of a typical AlN:Er sample deposited (**a**) without substrate heating and (**b**) with substrate temperature maintained at 400 °C.

The surface and cross-sectional morphology of the AlN:Er films were observed by FESEM. The results are shown in Figure 5. The surface morphology of an AlN:Er sample (deposited at 400 °C) is shown in Figure 5a, which illustrates that the surface of the sample is uniform and smooth. Figure 5b,c is the cross-sectional views of a typical AlN:Er sample deposited without intentional-heating and with substrate temperature maintained at 400 °C, respectively. It is illustrated that both of these two samples show similar dense and compact columnar crystalline structures, and no apparent difference could be identified. The reason for this kind of crystalline growth is due to the fact that increased ion to neutral flux ratio has been obtained from our unbalanced magnetron deposition system [33], and therefore the sputtered adatoms on the substrate have enough kinetic energy or high probability to diffuse and rearrange themselves to construct the dense and compact crystalline structures.

### 3.3. Surface Roughness

**Figure 6:**
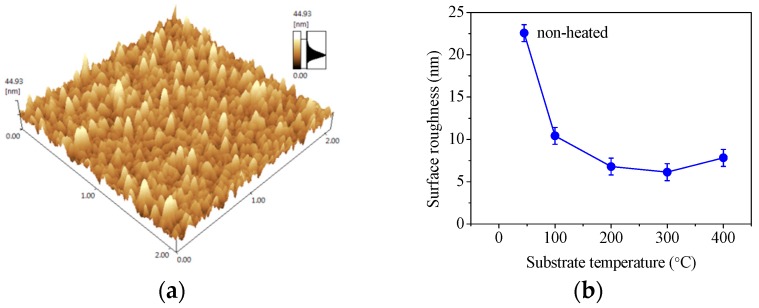
(**a**) Atomic force microscopy (AFM) image of an AlN:Er film deposited at 200 °C; (**b**) dependence of AlN film surface roughness on substrate temperature.

Surface roughness is an important parameter of optical thin films such as AlN:Er. To apply the luminescence sensing technique, a smooth surface with less scattering is desirable for the detection of luminescence signal from the sample surface. The substrate temperature dependence of the surface roughness of the AlN:Er films was investigated via AFM measurements. A typical AFM image of an AlN:Er film sample (deposited at 200 °C) is shown in Figure 6a, and the surface root-mean-square (RMS) roughness was calculated from the entire scanned region.

The RMS surface roughness of the deposited AlN:Er films was plotted against the substrate temperature in Figure 6b. It is shown clearly that, generally, higher substrate temperature leads to lower surface roughness. The AlN:Er sample deposited without intentional heating has the largest RMS roughness of 22.6 ± 1.0 nm, while the AlN:Er sample deposited at 300 °C has the lowest RMS roughness of 6.1 ± 1.0 nm. The reason for these could be attributed to the crystalline structure evolution process, which is discussed in Section 3.4. Surface roughness could also be one of the reasons for lower photoluminescence intensity from the samples deposited at lower substrate temperature.

### 3.4. Crystalline Structure

**Figure 7:**
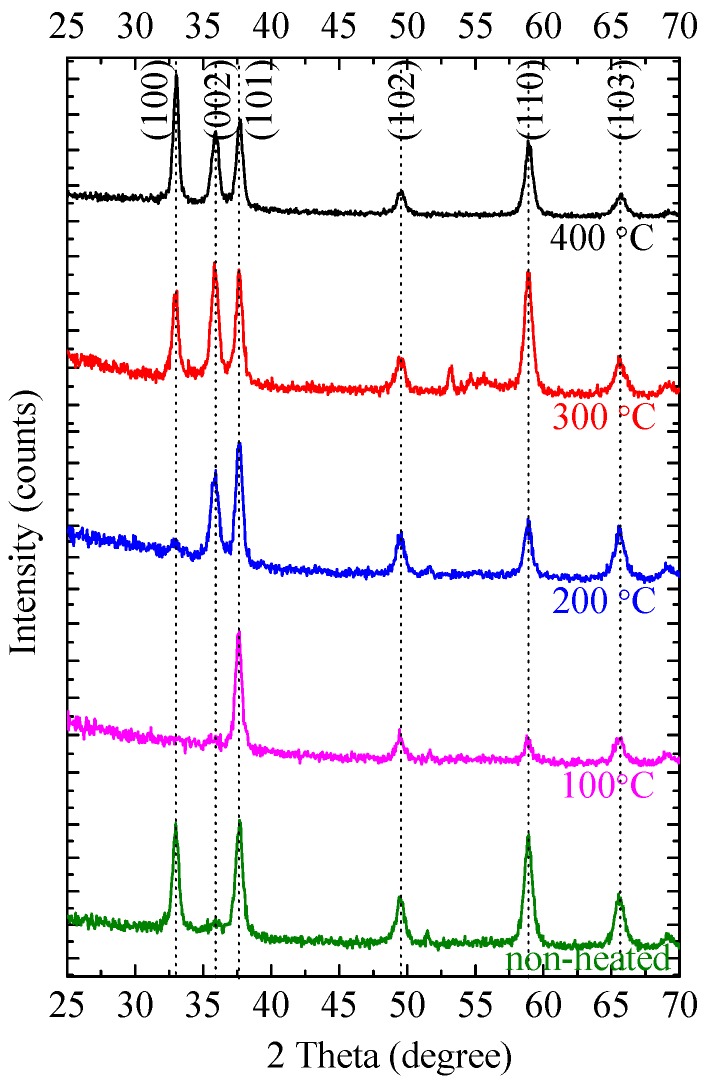
Grazing incidence X-ray diffraction (GIXRD) pattern of AlN:Er films prepared at various substrate temperatures. Note that the spectra have been shifted vertically for clarity purposes.

The crystalline structure of the AlN:Er films deposited at different substrate temperatures were measured by GIXRD and the measured patterns are illustrated in Figure 7. The reflection peaks were indexed using the standard inorganic crystal structure database pattern (ICSD PDF#89-3446) of hexagonal wurtzite AlN. It is illustrated in Figure 7 that all the AlN:Er films were polycrystalline, and the major peaks correspond to the (100), (002), (101) and (110) orientations.

The results shown in Figure 7 apparently illustrate that the substrate temperature has a profound influence on the major reflection peaks of the AlN:Er films. However, the dependence of these major reflection peaks on the substrate temperature shows no simple rule. We find that this phenomenon could be understood via the study of the crystallographic unit lattice of hexagonal wurtzite AlN.

**Figure 8:**
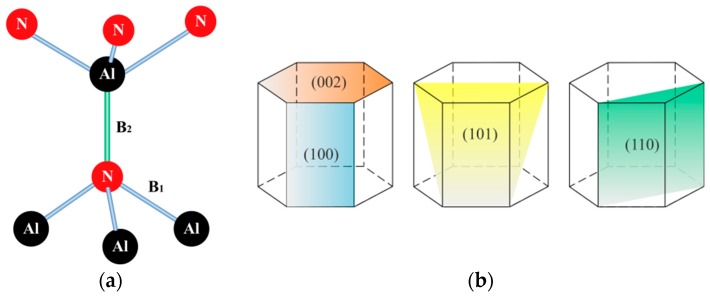
(**a**) Al–N bonds formed distorted tetrahedron and (**b**) geometry of crystallographic planes for (100), (002), (101) and (110) in hexagonal AlN lattice. (After Reference [34]).

In the unit lattice of hexagonal AlN, each Al atom is bonded with four N atoms and forms a distorted tetrahedron with two kinds of Al–N bond, as shown in Figure 8a [34]. Each of the three N atoms in the plane perpendicular to the c-axis form equivalent Al–N bonds with the central Al atom, and this is the first kind of Al–N bond, denoted as the B_1_ bond shown in Figure 8a; whereas another kind of Al–N bond is formed in the direction parallel to the c-axis, denoted as the B_2_ bond shown in Figure 8a. Additionally, the bond lengths of the B_1_ and the B_2_ bond are 0.1885 nm and 0.1917 nm, respectively. As the bond length of the B_1_ bond is shorter than the B_2_ bond, higher energy is needed to form the B_1_ bond, or simply, the formation energy of the B_1_ bond is higher than the B_2_ bond [34].

The crystallographic planes for (100), (002), (101) and (110) in the hexagonal AlN lattice are shown in Figure 8b. It is clearly illustrated that the (100) and (110) planes only contain the B_2_ bond and with loosely packed equal numbers of Al and N atoms, while the (002) plane only contains the B_1_ bond with closely packed either all Al atoms or all N atoms (note that the lattice parameters of hexagonal wurtzite AlN are *a* = *b* = 3.123 Å and *c* = 4.988 Å, ICSD PDF#89-3446). Additionally, the (101) plane contains both the B_1_ and the B_2_ bond. As the formation energy of the B_1_ bond is higher than the B_2_ bond, the (002) plane has the highest formation energy, the (100)/(110) plane has the lowest formation energy, and the (101) plane has the medium formation energy.

The evaluation process of the GIXRD patterns shown in Figure 7 can now be understood as follows: For non-heated substrate, the sputtered adatoms on the substrate did not have enough kinetic energy, thus the low formation energy planes, i.e., the (100), (101) and (110) planes established; as the substrate temperature rose to 100 °C, the adatoms arrived on the substrate got larger surface mobility, so the medium formation energy plane, i.e., the (101) plane, was developed; further increasing the substrate temperature to 200 °C led to the emergence of another peak at (002) reflection, this is due to the fact that the adatoms now had sufficient kinetic energy to form the closely packed (002) plane; at 300 °C the (100) reflection appeared again, this is due to the fact that at this high temperature level, the adatoms had more sufficient high kinetic energy, and therefore they started to rearrange themselves to the low formation energy planes such as (100) before the next layer of adatoms arrive. This is also evidenced by the enhancement of the (110) reflection, which also belongs to the low formation energy plane; further rising the substrate temperature to 400 °C still exhibited such kind of rearrangement of the high kinetic energy adatoms to the low energy planes, such as the (100), (101) and (110) planes, also note that the (100) reflection became relatively stronger even than the (002) and (110) reflections. A similar evolution process for pure AlN films deposited with substrate temperature maintained at 100~500 °C has also been observed by Cheng et al. [22], but little explanation of the evolution process was given.

Hence, the kinetic energy of the sputtered adatoms has a profound influence on the major reflection peaks of the deposited AlN:Er films, and substrate temperature is an effective control method to obtain AlN:Er films with desired crystalline structure, which has a significant effect on their optical and mechanical properties. The surface roughness dependence on the substrate temperature shown in Figure 6b could be now understood readily: At higher substrate temperature, the rearrangements of the adatoms fills the gap between the columnar crystalline structures, thereby smoothing the film surface.

Based on the GIXRD patterns, we performed the Williamson-Hall analysis [35] to estimate the strain and crystallite size of the polycrystalline AlN:Er films. The intention of this is because that the dielectric function, i.e., the index of refraction and coefficient of extinction, has strong dependence on the strain and crystallite size of the deposited AlN:Er films [36]. The strain and the crystallite size of the AlN:Er films could be estimated by using:

(1) $$\frac{\beta \cos\theta }{\lambda }=\frac{1}{D}+\frac{4\varepsilon \sin\theta }{\lambda }$$

where *β* is the full width at half maximum in radians, *θ* is the diffraction angle, *λ* is the wavelength of the X-ray, *D* is the crystallite size and *ε* is the strain. We plotted *β*cos*θ*/*λ* versus sin *θ*/*λ* for the AlN:Er films deposited under different substrate temperature conditions in Figure 8, and then the dotted data were linearly fitted. The slope of the fitting line gives four times the estimated strain, and the y-intercept gives the inverse of the estimated crystallite size.

**Figure 9:**
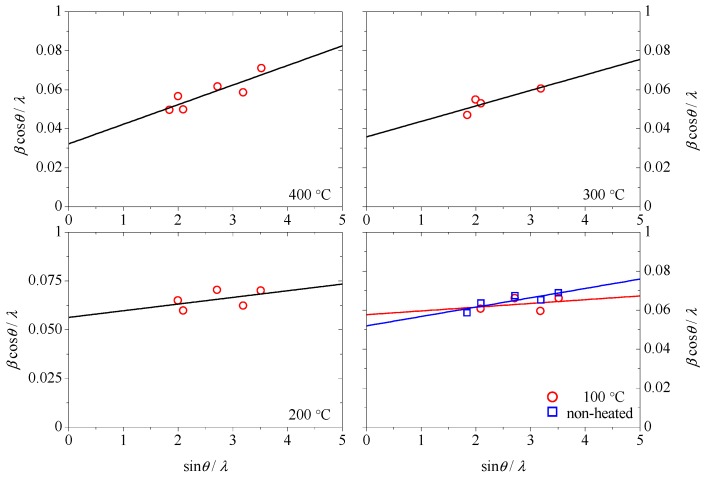
Williamson-Hall analysis of the X-ray diffraction patterns of the AlN:Er films deposited under different substrate temperatures conditions.

**Figure 10:**
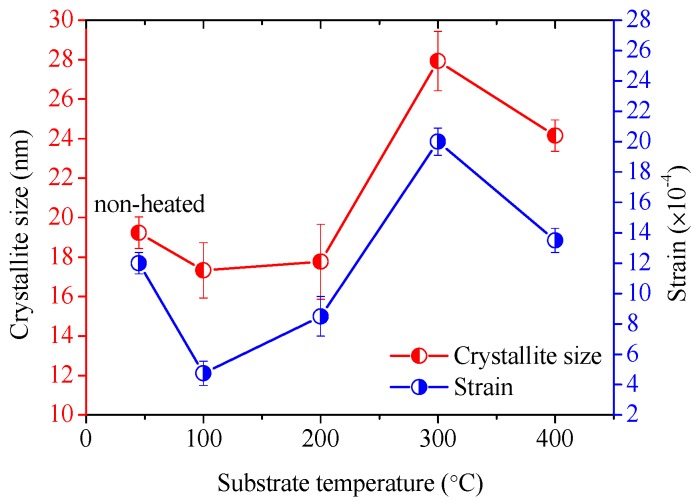
Dependence of average strain and crystallite size on the substrate temperature.

Based on the Williamson-Hall analysis illustrated in Figure 9, the average strain and average crystallite size of the deposited AlN:Er films were obtained simultaneously, and the results are plotted against the substrate temperature in Figure 10.

It is illustrated in Figure 10 that both the average strain and crystallite size have similar behavior as the substrate temperature varies. Generally, the AlN:Er films deposited under lower substrate temperatures show smaller average crystallite size and lower level of average strain. The AlN:Er film deposited at 300 °C has the largest crystallite size of ~28 nm, compared to 10~18 nm for the AlN:Er films deposited at lower substrate temperatures or non-heated. The decrease of the average crystallite size when the substrate temperature increases from 300 to 400 °C might be due to reduced thickness of the AlN:Er film [37], as discussed in Section 3.5. These average crystalline size and strain results are similar to the results reported in the literature [38,39,40].

In comparison to the AlN:Er films deposited under other substrate temperature conditions, the one deposited at 300 °C also exhibits the largest strain. This might be due to the sufficient surface diffusion and rearrangements of the adatoms on the substrate surface at such high temperature. However, both the crystallite size and average strain for the AlN:Er films deposited at 400 °C show lower values than its 300 °C counterpart. The possible reason for this could be the further diffusion and rearrangements of the adatoms to the loosely-packed or low formation energy planes, i.e., the (100) and (101) planes. This is evidenced by the increase of the relative diffraction intensities of the (100) and (101) reflections, and the decrease of that for the (002) reflection, when the substrate temperature rose from 300 to 400 °C.

### 3.5. Optical Properties

The film thickness and the dielectric function of the absorbing AlN films were obtained via VASE measurements. A Lorentz oscillator model was used to model the dielectric function of the AlN:Er film [36]. Then, the modeled ellipsometric parameters were fitted with the measured results. Upon fitting, the film thickness, the refractive index and the extinction coefficient of the AlN:Er film were obtained concurrently. The quality of fitting was denoted by the mean squared error, which was less than 3 for all of our fittings.

**Figure 11:**
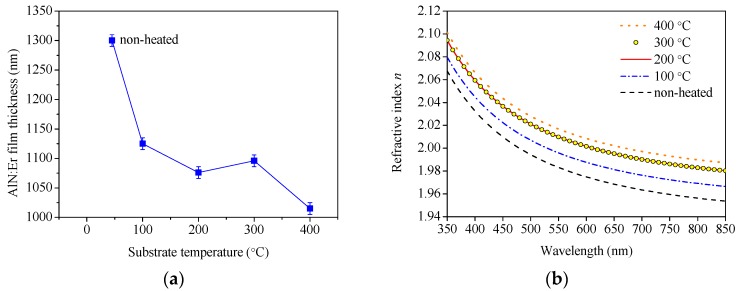
Dependence of AlN:Er film (**a**) thickness and (**b**) refractive index on the substrate temperature.

The dependence of the AlN:Er film thickness on the substrate temperature is plotted in Figure 11a. The AlN:Er film deposited without intentional substrate heating has the largest thickness of ~1300 ± 10 nm, while the 400 °C counterpart has the smallest thickness of ~1015 ± 10 nm. The refractive index of the AlN:Er films deposited under different substrate temperature conditions are shown in Figure 11b. Note that all the revealed extinction coefficient results are less than 1 × 10^−3^ and not shown here. We can see from Figure 11b that the index of refraction of the AlN:Er films increases gradually while the substrate temperature increases from non-heated to 400 °C. Note that the difference between the 200 and 300 °C results are quite small.

The evolution of the thickness of the deposited AlN:Er film could be attributed to two aspects: At elevated substrate temperature conditions the adatoms diffuse and rearrange themselves to fill the gap between the columnar crystals, thus the thickness of the AlN:Er films obtained under higher substrate temperatures should be smaller, and the film structure should be denser and more compact, which means larger index of refraction; high temperature substrate could also facilitate desorption processes of the adatoms with lower kinetic energies and incapable to satisfy the thermodynamic conditions at the growing surface [41]. More adatoms might be desorbed from the substrate maintained at higher temperature, so the thickness of the deposited film decreases with the increase of the substrate temperature.

### 3.6. Photoluminescence

**Figure 12:**
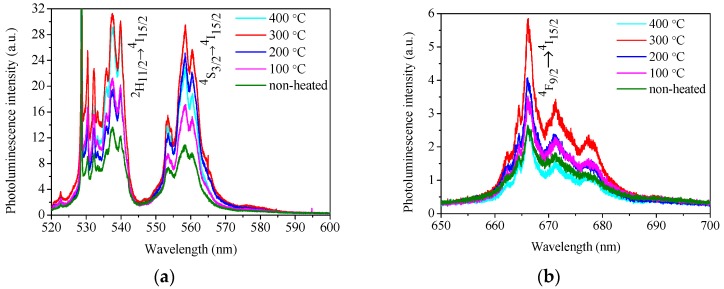
Photoluminescence spectra of AlN:Er films deposited at various substrate temperatures (**a**) the main peak locates around 540 nm and 560 nm corresponds to the ^2^H_11/2_→^4^I_15/2_ and the ^4^S_3/2_→^4^I_15/2_ electronic transitions of Er^3+^ ions, respectively and (**b**) the weak emission peak locate around 670 nm corresponds to the ^4^F_9/2_→^4^I_15/2_ transition.

The recorded PL spectra of the AlN:Er films deposited under different substrate temperature conditions are plotted in Figure 12, where the main peaks are denoted by the Russell-Saunders notations for the related electronic transitions of Er^3+^ ions [42]. Note that the AlN:Er films were prepared without further post-deposition thermal treatment. It is seen from Figure 12a that all the AlN:Er films exhibit the featuring emission peaks of Er^3+^ ions near 540 nm and 560 nm, which corresponds to the ^2^H_11/2_→^4^I_15/2_ and the ^4^S_3/2_→^4^I_15/2_ electronic transitions of Er^3+^ ions, respectively. The ~530 nm peak is due to the Raman scattering from the crystalline Si substrate (see, for example, Reference [43]). As shown in Figure 12b, there is also a weak emission peak around 670 nm, which corresponds to the ^4^F_9/2_→^4^I_15/2_ transition.

**Figure 13:**
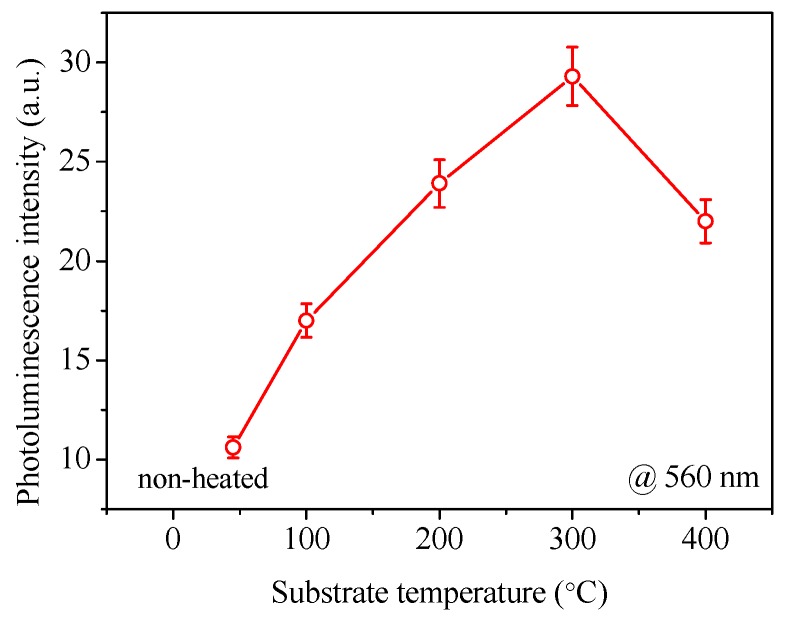
Dependence of photoluminescence intensity (around 560 nm) of AlN:Er films on the substrate temperature during film deposition.

The PL intensity of the AlN:Er films around 560 nm is plotted against the substrate temperature in Figure 13. We can see that, as the substrate temperature increases from non-heated condition to 300 °C, the PL intensity increases monotonically. When the substrate temperature further increases to 400 °C, the PL intensity falls back. The reason for this phenomenon could be linked to two main factors: The refractive index and the crystallite size of the deposited AlN:Er films.

From Figure 11b, generally, higher substrate temperature means higher index of refraction of the AlN:Er film. In addition, according to Fermi’s golden rule, the spontaneous emission rate of the excited Er^3+^ ion is proportional to the refractive index of its host medium [15]. Consequently, higher substrate temperature leads to higher intensity of PL emission.

The crystallite size is another factor that should be considered. The GIXRD patterns of the fabricated AlN:Er films, as illustrated in Figure 7, only exhibit the distinct peaks belonging to hexagonal wurtzite AlN, and no Er-related phases have been identified. This signifies that the Er ions are mostly embedded in the grain and grain boundaries of the AlN host and only take the substitute Al site of the wurtzite AlN lattice. The desired locations for the Er^3+^ ions are inside the grains, because the optical environment ‘seen’ by these ions, from the point of view of the photonic density of states, are better than their counterparts located on the grain boundaries. Additionally, larger crystallite size means that more Er^3+^ ions are in the desired optical environment. Based on the consideration of both the refractive index and the average crystallite size, the AlN:Er film deposited at 300 °C exhibits the highest PL intensity. Note that the film thickness might also influence the PL intensity, though we find this effect is limited.

## 4. Conclusions

Substrate temperature dependent properties of AlN:Er thin films prepared by reactive radio-frequency magnetron sputtering have been illustrated in this work. The XPS results signify that the fabricated AlN films were uniformly doped with Er^3+^ ions; the cross-sectional morphology observations via FESEM illustrates that the AlN:Er films were made of dense and compact columnar structures, regardless of the substrate temperature concerned in this work; the surface morphology investigation via AFM reveals that the RMS roughness is merely ~6.1 ± 1.0 nm for the AlN:Er films deposited at 300 °C, compared to the largest roughness of 22.6 ± 1.0 nm for the non-heated counterpart; the evolution of the reflection patterns obtained via GIXRD were explained via the study of the crystallographic unit lattice of hexagonal wurtzite AlN; the Williamson-Hall approach has been applied to the reflection patterns to obtain the average crystallite size, and the results indicate that the AlN:Er film deposited at 300 °C exhibits the largest crystallite size of ~28 nm, compared to 10~18 nm for the AlN:Er films deposited at lower substrate temperatures; the AlN:Er film deposited at 300 °C also shows the strongest room temperature PL intensity. These results obtained via different techniques were self-consistent and discussed in detail in the previous section.

This work demonstrates that substrate temperature has a significant influence on various properties of the luminescent AlN:Er films. It suggests that the optimal substrate temperature for the deposition of the AlN:Er film is 300 °C, if the PL intensity is the most important factor to be considered. However, at this substrate temperature, certain thin metal components might deform due to unbalanced heating at different locations. Then, the lower substrate temperatures such as 100 °C or even non-intentional heating might also be adopted, though weaker PL intensity would be anticipated.
